# *WEE1* kinase polymorphism as a predictive biomarker for efficacy of platinum-gemcitabine doublet chemotherapy in advanced non-small cell lung cancer patients

**DOI:** 10.1038/srep11114

**Published:** 2015-06-09

**Authors:** Di Liu, Chunyan Wu, Yuli Jiao, Likun Hou, Daru Lu, Hui Zheng, Chang Chen, Ji Qian, Ke Fei, Bo Su

**Affiliations:** 1 Central Laboratory; 2 Department of Pathology; 3 Department of Thoracic Surgery, Shanghai Pulmonary Hospital, Tongji University School of Medicine; 4 Fudan University Shanghai Cancer Center, State Key Laboratory of Genetic Engineering and MOE Key Laboratory of Contemporary Anthropology, School of Life Sciences, Fudan University, Shanghai, P.R. China

## Abstract

DNA-damaging agents are commonly used for first-line chemotherapy of advanced non-small cell lung cancer (NSCLC). As a G2/M checkpoint kinase, Wee1 can phosphorylate CDC2-tyr15 and induce G2/M cell cycle arrest in response to DNA damage. The correlation of *WEE1* polymorphisms to the efficacy of chemotherapy was tested in 663 advanced NSCLC patients. *WEE1* rs3910384 genotype correlated to overall survival (OS) and progress-free survival (PFS) of NSCLC patients treated with platinum-based chemotherapy. Sub-group analysis revealed that rs3910384 was particularly associated with the efficacy of doublet chemotherapy combining two DNA-damaging agents, i.e. platinum and gemcitabine. NSCLC patients with the *WEE1* rs3910384 G/G homozygote genotype showed 13.5 months extended OS, 3.2 months extended PFS, and a 274% relative increase in their 3-year survival rate (from 7.4% to 27.7%) compared to the A/A+A/G genotype after treatment with platinum-gemcitabine regimen. This finding was reproduced in the validation cohort. We utilized a luciferase reporter assay and Electrophoretic Mobility Shift Assay (EMSA) to demonstrate that rs3910384-linked *WEE1* promoter haplotype can mediate allele-specific transcriptional binding and *WEE1* expression in DNA damage response. In conclusion, the *WEE1* rs3910384 G/G homozygote genotype can be used as a selective biomarker for NSCLC patients to indicate treatment with platinum and gemcitabine regimen.

Lung cancer remains the most dangerous malignant disease, with the highest incidence and mortality throughout the world as well as in China^1^,^2^. Non-small-cell lung cancer (NSCLC) accounts for approximately 80% of lung cancer cases and most patients are diagnosed at an advanced stage^3^. Targeted therapy is already being used for the treatment of advanced NSCLC harboring *EGFR* mutations, *EML4-ALK* fusion, *etc*^4^. However, the patients with *EGFR* mutations only account for 10–35 percent of NSCLC patients and the frequency of *EML4-ALK* fusion patients is just 5–7 percent^5^, meaning that the remaining patients still need to rely on platinum-based chemotherapy. Although the platinum-based doublet chemotherapy is the most common first-line treatment for patients with advanced NSCLC, the efficacy of the chemotherapy regimen remains far from ideal and patients usually experience severe adverse effects. As a result, fewer than 35% of patients show a positive response to platinum-based chemotherapy and the 3-year survival rate is less than 10% in unselected NSCLC patients^6^.

DNA-damaging agents, such as platinum, gemcitabine, and 5-fluorouracil (5-FU), are the primary chemotherapy drugs used to treat many cancers. These drugs cause cell cycle arrest by impeding DNA synthesis, replication, and transcription, to eventually induce cancer cell apoptosis or mitotic catastrophe^7^,^8^. For lung cancer, platinum agents are indispensable in the standard chemotherapy doublet regimens. Evidence-based clinical practice shows that two-drug combinations are an optimal chemotherapy regimen for the treatment of advanced NSCLC. The combinations of cisplatin or carboplatin with either one of the DNA-damaging agents (gemcitabine), tubulin-targeting drugs (paclitaxel, docetaxel, or vinorelbine), or DNA topoisomerase II inhibitors (VP-16), show almost similar response rates and survival time for unselected NSCLC^9^. Therefore, clinically applicable biomarkers for prediction of the efficacy of combination chemotherapy are urgently needed for personalized chemotherapy treatment of NSCLC.

Wee1, a crucial kinase of cell cycle progression, regulates the G_2_ checkpoint arrest in response to DNA damage. Wee1 can phosphorylate CDC2 (CDK1) on Tyr15, inactivating the CDC2-cyclin B complex to inhibit cell cycle progression from G_2_ into M phase^10^. DNA damage checkpoints can induce a transient increase of *WEE1* expression to cause G_2_-arrest for premitotic DNA repair^11^. Wee1-induced G_2_-arrest plays a critical role in the sensitivity of DNA-damaging agents and/or radiation therapy^12^. The combination of DNA-damaging drugs or radiation therapy with *WEE1* kinase inhibitors has shown therapeutic benefit^14^. In this study, we find that *WEE1* polymorphism is strongly associated with the treatment efficacy of DNA-damaging agents in patients with advanced NSCLC and can be used as a valuable biomarker for predictions of efficacy followed by a platinum-gemcitabine regimen.

## Materials and Methods

### Ethics Statement

The study was approved by the Medical Ethics Committee of the Shanghai Chest Hospital, Shanghai Zhongshan Hospital, Shanghai Changhai Hospital, and Shanghai Pulmonary Hospital. Written informed consent was obtained from all patients who participated in this study. In addition, the experiments in this study were conducted in accordance with approved guidelines and regulations.

### Study subjects

The test cohort included 663 patients who had been diagnosed with either clinical stage IIIA or IV NSCLC and had received first-line platinum-based chemotherapy (no prior surgery, radiotherapy, or concurrent chemoradiotherapy) between March 2005 and January 2010. The association of the WEE1 tag SNP with the efficacy of chemotheraputic regimens was reviewed and evaluated. The validation cohort included 264 NSCLC patients who accepted the platinum-gemicitabine regimen in Shanghai Pulmonary Hospital between June 2010 and May 2013. All the patients had histologically confirmed NSCLC with the presence of at least one measurable and evaluable lesion. Eastern Cooperative Oncology Group performance status (ECOG PS) for the patients is 0–2 and the cardiovascular, hepatic, hematologic, and renal functions of the patients have been reviewed to assess chemotherapy tolerance.

### Treatment

All patients in the test cohort underwent first-line platinum-based chemotherapy for 2 to 6 cycles with one of the following chemotherapy doublet regimens: (1) DNA-damaging agents regimen: either cisplatin 75 mg/m^2^ or carboplatin AUC 5 administered on day 1 every 3 weeks, in combination with gemcitabine 1250 mg/m^2^ on days 1 and 8 every 3 weeks, (2) tubulin-targeting drugs regimens: Either cisplatin 75 mg/m^2^ or carboplatin AUC 5 administered on day 1 every 3 weeks, in combination with navelbine 25 mg/m^2^ on days 1 and 8 every 3 weeks, or paclitaxel 175 mg/m^2^ on day 1 every 3 weeks, or docetaxel 75 mg/m^2^ on day 1 every 3 weeks, (3) other combination: Either cisplatin 75 mg/m^2^ or carboplatin AUC 5 administered on day 1 every 3 weeks, in combination with etoposide or bevacizumab. For the patients in validation cohort, either cisplatin 75 mg/m^2^ or carboplatin AUC 5 was administered on day 1 every 3 weeks, in combination with gemcitabine 1250 mg/m^2^ on days 1 and 8.

### Tumor response

According to the Response Evaluation Criteria in Solid Tumors (RECIST), the short-term responses to chemotherapy treatment were evaluated after the first 2 cycles. The disease control rate (DCR) included complete response (CR), partial response (RR) and stable disease (SD). The objective response rate (ORR) consisted of complete response (CR) and partial response (PR). The progression-free survival (PFS) was assessed as the date from the chemotherapy treatment to objective disease progression or death (whichever occurred first) or the last progression-free follow-up. The overall survival (OS) was defined as the first day to receive chemotherapy treatment to the day of death or to the final follow-up.

### *WEE1* tag SNPs genotyping and Linkage Disequilibrium Analysis

The four tag SNPs (rs3829254, rs3910384, rs4370932 and rs1049403) representing 50 SNPs of *WEE1* were selected from the CHB data of the phase 2 HapMap SNP database (http://www.hapmap.org/) using a minor allele frequency (MAF) cutoff of 0.05 and a correlation coefficient (r^2^) threshold of 0.8. Genomic DNA was extracted from patients’ peripheral blood samples using the QIAamp DNA Maxi Kit (Qiagen GmbH, Hilden, Germany) according to the manufacturer’s instructions. The SNPs were genotyped using the iSelect HD Bead-Chip (Illumina, San Diego, CA, USA) with the following quality-control criteria: genotyping call rate >0.95; MAF >0.05; and GenCall score >0.2. The concordance among replicates was >99.9% and the GeneMap software was used to analyze the data and prepare reports.

Linkage disequilibrium was analyzed in Haploview Software (Broad Institute of Harvard and Massachusetts Institute of Technology, Cambridge, MA, USA). Three putative functional SNPs, *i.e*. rs6486433, rs3763869 and rs3763868 in the *WEE1* promoter region which are closely linked with rs3910384 were selected using MAF >0.2 and D’ >0.8 cutoff. The function of SNPs was predicted with FuncPred tools (National Institute of Environmental Health Sciences, USA). Individual haplotype frequencies for the selected SNPs were evaluated using the Hyploview or PHASE 2.0 Program (version 2.0.2) based on the Bayesian algorithm.

### Construction of PGL4 *WEE1* luciferase reporter plasmids and luciferase reporter Assay

Genomic DNA was extracted from the peripheral blood lymphocytes of patients. The *WEE1* haplotype promoter region (650 bp;−24 bp ∼+626 bp) containing the 3 putative functional SNPs was PCR-cloned into PGL4 luciferase reporter plasmid. Forward primer: 5’-CACGGTACCAGCCAGTGTCCAGCCTAAGCA-3’ (Kpn I); Reverse primer: 5’-CTAACGCGTAGGTCTCCTCAGGTCCAGTCTCA-3’ (Mlu I). The PCR reaction was carried out at 95 °C, 3 minutes followed by 35 cycles (95 °C 30 s, 68 °C 30 s, 72 °C 30 s) and an extension period of 5 minutes at 72°C. PCR products were ran on an agarose gel and purified from the bands (MiniBEST Agarose Gel DNA Extraction Kit, TaKaRa, China), ligated, and transformed into DH5α. PGL4 *WEE1* luciferase reporter plasmids containing two major promoter haplotypes, *i.e*. rs3910384 “A” allele-linked G/A/C (rs6486433/rs3763869/rs3763868) (termed “haplotype 1”) or rs3910384 “G” allele-linked T/G/G (rs6486433/rs3763869/rs3763868) (termed “haplotype 2”) were obtained by sequencing of the selected single clones.

The PGL4 *WEE1* luciferase reporter and TK plasmid (DNA dosage PGL-4: TK = 9:1) were co-transfected into A549 or H1299 (human lung adenocarcinoma) cell lines using Lipo FiterTM liposomal Transfection regent (Hanbio, China). After the indicated time, the cells were lysed, and relative luciferase units (RLU) were measured using the dual-luciferase reporter assay system (Promega, WI, USA) according to the manufacturer’s instructions. To determine the *WEE1* RLU after DNA damaging agents treatment, the transfected cells were subjected to 8 μg/mL cisplatin (DDP) or 40 μg/mL gemcitabine (GEM) for two hours and RLU was measured at 12, 24, and 36 hours after drug removal. Each assay was repeated at least three times.

### Electrophoretic Mobility Shift Assay

Nuclear extracts of lung cancer cell lines H1299 and A549 were prepared using the method described by Edmead *et al*^15^. The sequences of the biotin-labeled double-stranded oligonucleotide probes of rs6486433, rs3763869 and rs3763868 are listed in Table S1. EMSA was carried out according to the manufacturer’s protocol (EMSA Kit ES009, Beyotime, China). Briefly, 10 μg nuclear extract were incubated in 20 μl binding reaction buffer (10 mM Tris-HCl, pH7.5, 50 mM KCl, 1 mM DTT, 10% glycerol) with the 2.5 nM biotin-labeled probe and 0.5 μg poly dI:dC (Sigma) for 30 min at room temperature. DNA–protein complexes were subjected to electrophoresis on 5% polyacrylamide gel, transferred to nylon membrane, and visualized by chemiluminescence imaging.

### Statistical analysis

All statistical analyses were accomplished using SPSS® version 20.0 (SPSS Inc., Chicago, IL, USA). The association between OS or PFS of the NSCLC patients and the tag SNPs was evaluated by the Kaplan-Meier curves and verified by log-rank tests. Univariate and multivariate analyses were executed utilizing the Cox proportional hazard model to validate the significant factors related to the OS or PFS. Variables yielding *P*-values < 0.05 in the univariate analyses were used for the multivariate analyses, including gender (female *vs*. male), smoking status (ever *vs*. never), clinical stage (TNM IV *vs*. IIIb *vs*. IIIa), histology (adenocarcinoma, squamous, adenosquamous or others), the rs3910384 dominant model, the rs3829254 recessive model, and the rs4370932 additive model. For the dominant, recessive and additive models of each SNP, the model with the smallest *P*-value in univariate analyses was used for multivariate analyses. The relationship between the significant SNPs and the clinical characteristics was determined using χ^2^ test. All statistical analyses were two-sided and the differences were considered as statistically significant at *P* < 0.05.

## Results

### Patients’ characteristics and genotype information

The details of the patients’ characteristics in the test cohort are listed in Table S2. All 663 patients were treated with first-line platinum-based doublet chemotherapy in this study. Among them, 152 patients received the platinum-DNA damaging drug combination regimen (gemcitabine), 472 patients received the platinum-tubulin-targeting drug combination regimen (paclitaxel, docetaxel or navelbine), and the rest received the other combination regimen, *i.e*. etoposide or bevacizumab. The patient population consisted of 465 males and 198 females with a median age of 58 years. The tumors were classified histologically as 64.9% adenocarcinomas, 21.3% squamous carcinomas, 2% adenosquamous carcinomas, and 11.9% other histological types.

The genotype frequencies of the four *WEE1* tag SNPs in current data, or in CHB (Han Chinese in Beijing, China), CEU (Utah residents with Northern and Western European ancestry from the CEPH collection), and YRI (Yoruban in Ibadan, Nigeria) from the HapMap (HapMap Genome Browser release #28; Phases 1, 2 & 3 - merged genotypes & frequencies) SNP database are shown in Table S3. No difference in allele frequency was found between the current data and CHB. Different rs3829254, rs3910384, rs4370932, rs1049403 allele frequencies were observed among the Chinese, Western and African population. The Rs3910384 MAF for Chinese, Western, and African populations were 0.401 (A), 0.411 (G), and 0.417 (A), respectively.

### *WEE1* rs3910384 genotype is correlated with the efficacy of platinum-based chemotherapy

**Figure 1:**
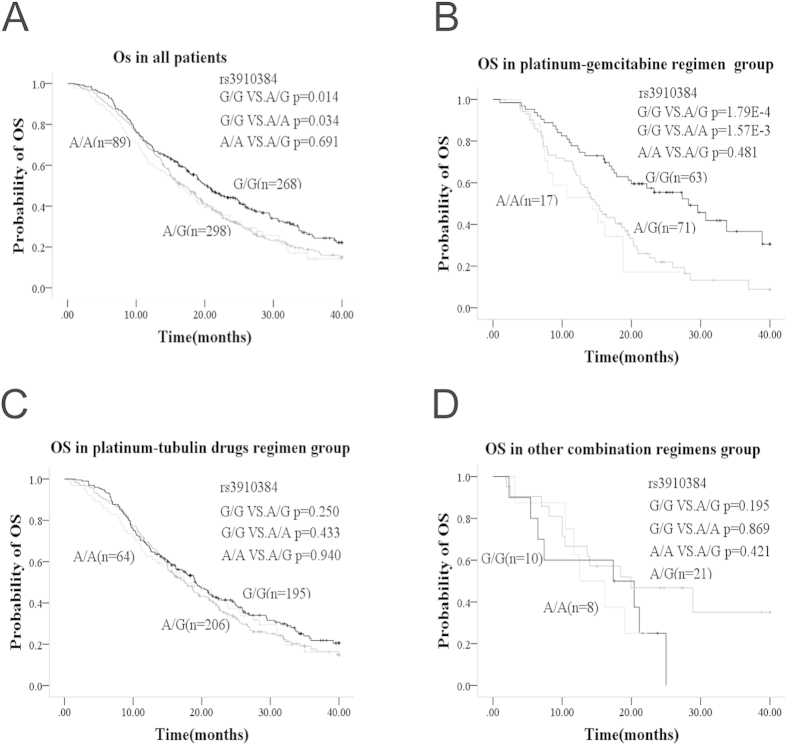
Kaplan-Meier overall survival curves of NSCLC patients with various rs3910384 genotypes treated with standard chemotherapy doublet regimens **A**: Overall survival for all the patients. **B**: Overall survival for patients treated with platinum-gemcitabine regimen. **C**: Overall survival for patients treated with platinum-tubulin-targeting drugs regimen. **D**: Overall survival for patients treated with other combination regimens.

The association of patients’ characteristics and *WEE1* polymorphisms with overall survival was explored by univariate Cox’s regression analysis in all 663 patients treated with platinum-based doublet chemotherapy (Table 1). The 4 *WEE1* tag SNPs were classified by models including genotypic, dominant, recessive, or additive. Gender, smoking history, clinical stage, and histology were significantly associated with OS but no correlation was found with age, ECOG PS, or chemotherapy regimens. Using *WEE1* genotypes as categorical variables, only rs3910384 in the 4 tag SNPs presented a statistically significant association with OS (*p* = 0.022). The dominant model of rs3910384 (*p* = 0.006), recessive model of rs3829254 (*p* = 0.022), and additive model of rs3910384 (*p* = 0.009), rs3829254 (*p* = 0.023), or rs4370932 (*p* = 0.028) showed statistically significant association. Furthermore, we included gender, smoking, clinical stage, histology, the rs3910384 dominant model, the rs3829254 recessive model, and the rs4370932 additive model in the multivariate Cox’s regression analysis. The results suggested that clinical stage (*p* = 8.0E-3), histology (*p* = 4.3E-4) and the rs3910384 dominant model (*p* = 4.0E-3) were independent predictive factors for OS (Table 2).Patients with the rs3910384 G/G genotype (mOS, 20.4; 95%CI, 17.6–23.2) had higher median OS (mOS) than A/G+A/A (mOS, 16.9; 95%CI, 15.1–18.7; *p* = 4.0E-3) in all 663 patients treated with platinum-based doublet regimens. The Kaplan-Meier curves of OS according to polymorphisms of the *WEE1* tag SNPs are shown in Fig. 1 & S1. (Fig. 1A for rs3910384; Figure S1A, B & C for other tag SNPs; Table S9).

**Figure 2:**
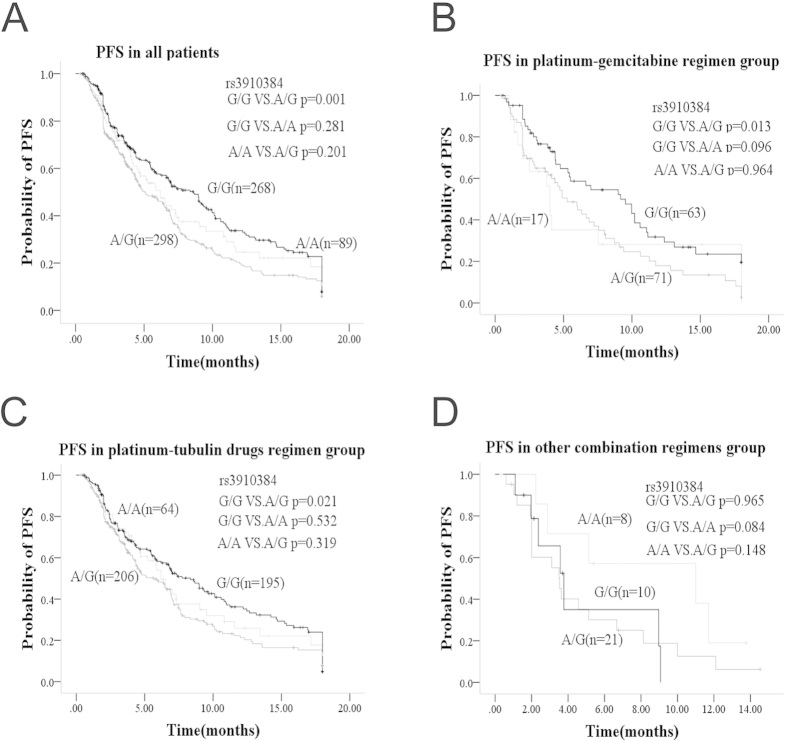
Kaplan-Meier progression free survival curves of NSCLC patients with variousrs 3910384 genotypes treated with standard chemotherapy doublet regimens **A**: Progression free survival for all the patients. **B**: Progression free survival for patients treated with platinum-gemcitabine regimen. **C**: Progression free survival for patients treated with platinum-tubulin-targeting drugs regimen. **D**: Progression free survival for patients treated with other combination regimens.

The *WEE1* rs3910384 dominant model (adjusted HR, 0.68; 95% CI, 0.55–0.84; *p* = 3.2E-4), as well as ECOG PS (adjusted HR, 1.49; 95% CI, 1.09–2.05; *p* = 0.012) were also found to be independent factors for the progression free survival (PFS) of all the enrolled NSCLC patients by univariate and multivariate Cox’s regression analysis (Table S4, Fig. 2A for rs3910384, Figure S2A, B & C for other tag SNPs).

### *WEE1* rs3910384 genotype predominantly contributes to the efficacy of platinum-gemcitabine regimen

To better define the contribution of *WEE1* rs3910384 to the efficacy of platinum-based chemotherapy, we performed a subgroup analysis stratified on different standard chemotherapy regimens and found that the rs3910384 genotype predominantly contributed towards the efficiency of platinum-gemcitabine regimen, which consists of two DNA- damaging agents, but contributed little to the efficacy of the other doublet regimens (tubulin-targeting agents or DNA topoisomerase inhibitors) used in the study (Fig. 1B–D; Table S5). The patients with *WEE1* rs3910384 G/G homozygote gained 32.2% (from 22.1% to 54.3%), 24.1% (from 14.7% to 38.8%) or 20.3% (from 7.4% to 27.7%) absolute increase in 1-, 2-, and 3-year-survival rates, respectively, compared to the A/A+A/G genotype (*p* = 3.5E-4) (Table S6). Subgroup multivariate Cox regression analysis revealed that *WEE1* rs3910384 dominate model and histology are independent factors for the OS of the 152 patients who received platinum-gemcitabine regimen (Table 3). The patients with G/G homozygote (mOS, 28.2; 95%CI, 20.5–35.9) had 13.5 months longer OS than A/A+A/G genotype (median OS, 14.7; 95%CI, 12.8–16.6; *p* = 6.9E-5). Adenocarcinoma patients were more sensitive than the squamous carcinoma, adenosquamous carcinoma, or other histological types when treated with platinum-gemcitabine regimen (*p* = 0.037). When evaluated with PFS as endpoint, a similar association in platinum-gemcitabine group was also observed, but not in the other platinum-based combination regimens (Fig. 2, Table S7).

For short-term response, NSCLC patients with the rs3910384 G/G genotype exhibited a higher disease control rate (DCR) following platinum-gemcitabine doublet treatment compared to A/A+A/G genotype patients with a marginal statistical significance (p = 0.058) (Table 4).

The results suggest that *WEE1* rs3910384 G/G genotype as a potential biomarker to predict the chemotherapeutic efficacy for the platinum-gemcitabine regimen of DNA-damaging agents and is valuable for personalized chemotherapy for advanced NSCLC patients.

### Validation of *WEE1* rs3910384 G/G to predict the efficacy of platinum-gemcitabine chemotherapy

**Figure 3:**
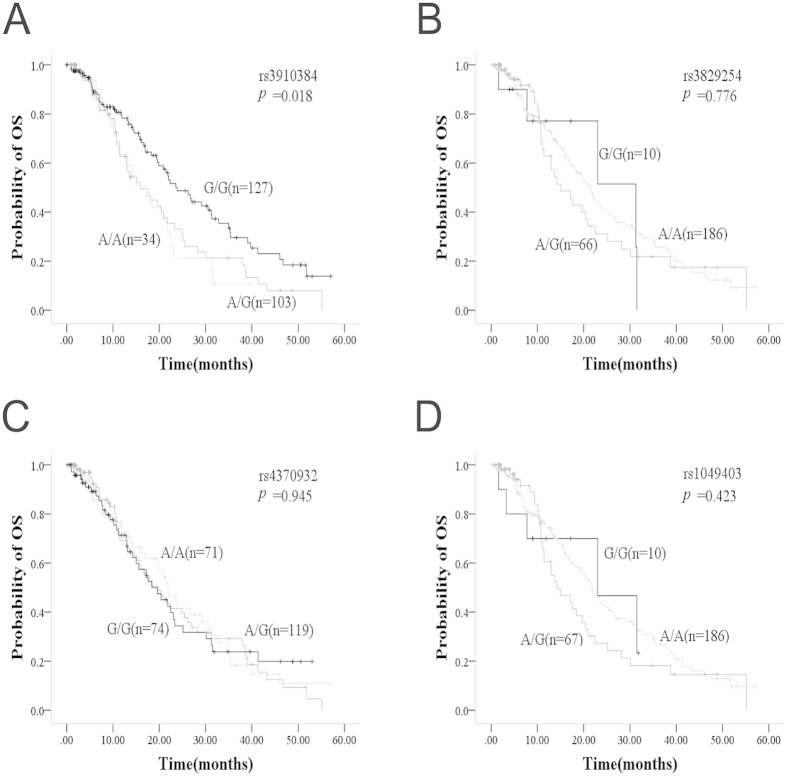
Validation of *WEE1* tag SNPs polymorphisms associated with the efficacy of DNA-damaging agents in 264 patients with advanced NSCLC **A**: Kaplan-Meier curve of OS for rs3910384 in patients with NSCLC. **B**: Kaplan-Meier curve of OS for rs3829254 in patients with NSCLC. **C**: Kaplan-Meier curve of OS for r4370932 in patients with NSCLC. **D**: Kaplan-Meier curve of OS for 1049403 in patients with NSCLC.

To validate the association of *WEE1* rs3910384 G/G with the efficacy of DNA-damaging agents, 264 patients with advanced NSCLC who received platinum-gemcitabine chemotherapy were subjected to *WEE1* rs3829254, rs3910384, rs4370932, and rs1049403 genotyping. The Kaplan-Meier analysis showed that *WEE1* rs3910384 was still significantly correlated with OS in the 264 patients (*p* = 0.018) rather than the other three tagSNPs, rs3829254, rs4370932 and rs1049403 (Fig. 3). Multivariant Cox regression analysis also suggested that *WEE1* rs3910384 dominate model was an independent factor for OS (*p* = 3.9E-4), which were consistent with findings in the test cohort (Table S8).

### Rs3910384 is closely linked with *WEE1* haplotype promoter with putative functional SNPs which can regulate *WEE1* transcription in DNA damage response

**Figure 4:**
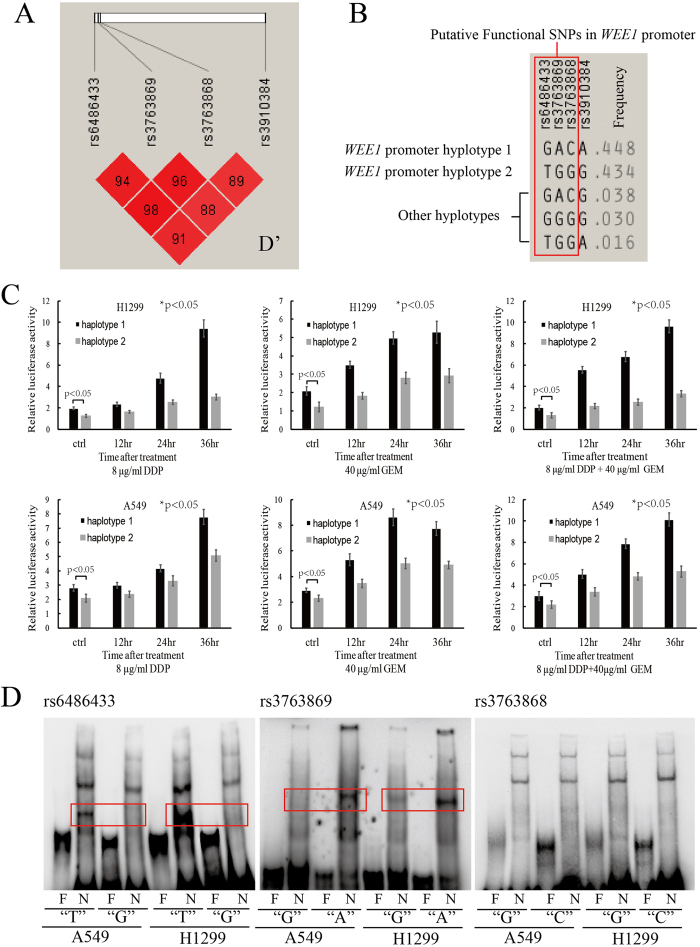
The allele-specific transcription regulation of WEE1 in NSCLC cell lines in DNA damage response by cisplatin and gemcitabine **A**: Linkage disequilibrium analysis of *WEE1* rs3910384 and putative functional SNPs in *WEE1* promoter region. **B**: *WEE1* promoter haplotypes containing the indicated putative functional SNPs. **C**: Dual luciferase reporter assay of *WEE1* haplotype promoters in A549 and H1299 cells following treatment with cisplatin or gemcitabine (*p < 0.05: repeated measures ANOVA; *p* < 0.05: *t*-test). **D**: EMSA for the 3 rs3910384-linked putative functional SNPs in *WEE1* promoter region.(**F**: free probe; N: nuclear extraction plus the indicated probe. Red frame indicates the emerged bands in contrast to the counterpart allele).

In an analysis of linkage disequilibrium using the data from Hapmap database, *WEE1* rs3910384 was found to be closely linked with the putative functional *WEE1* promoter at SNPs rs6486433, rs3763869 and rs3763868 (Fig. 4A). All 3 putative functional SNPs are located at the allele-specific transcriptional binding sites of the *WEE1* promoter region. The two major haplotypes of linked rs6486433/rs3763869/rs3763868/rs3910384 were G/A/C/A (44.8%) and T/G/G/G (43.4%) with the other haplotypes G/A/C/G (3.8%), G/G/G/G (3.0%), and T/G/G/A (1.6%) accounting for a small proportion of the total population (Fig. 4B). Thus the *WEE1* rs3910384 “A” allele is strongly linked to the putative functional rs6486433/rs3763869/rs3763868 (G/A/C) *WEE1* promoter and the rs3910384 “G” allele is strongly linked to rs6486433/rs3763869/rs3763868 (T/G/G).

Lower expression or functional loss of *WEE1* leads to higher sensitivity toward DNA-damaging agents^16^,^17^. Using the PGL4 *WEE1* haplotype promoter luciferase reporter assay, we confirmed that *WEE1* haplotype promoters can mediate allele-specific transcription of *WEE1* in lung cancer cell lines H1299 and A549 after treatment with the DNA-damaging chemotherapy agents cisplatin and gemcitabine (Fig. 4C). The *WEE1* promoter haplotype 2 with rs3910384 “G” allele-linked T/G/G (rs6486433/rs3763869/rs3763868) had lower RLU (relative luciferase activity) than the *WEE1* promoter haplotype 1 with rs3910384 “A” allele-linked G/A/C (rs6486433/rs3763869/rs3763868) at baseline levels in both A549 and H1299 cells (*p* < 0.05). Following DNA damage induced by cisplatin, gemcitabine, or the combination, the *WEE1* promoter haplotype 2 showed a much lower increase of RLU compared to the *WEE1* promoter haplotype 1 in A549 and H1299 lung cancer cell lines (*p* < 0.05). The results demonstrated that the *WEE1* promoter strength is weaker in haplotype 2 and stronger in haplotype 1 in the transcription and *de novo* synthesis of *WEE1* induced by the DNA damage response. The higher *WEE1* expression driven by promoter haplotype 1 (rs3910384 “A” allele-linked G/A/C) can result in resistance towards the DNA-damaging agents and the lower *WEE1* expression driven by promoter haplotype 2 (rs3910384 “G” allele-linked T/G/G) can result in sensitivity to DNA-damaging agents.

To examine the binding of SNP allele-specific transcriptional factors to Wee1 promoter haplotypes, we synthesized biotin-labeled probe pairs with the reference and allele genotypes of rs6486433, rs3763869, or rs3763868 and performed an electrophoretic mobility shift assay (EMSA) with the nuclear extract of A549 and H1299 cell lines. The results demonstrated that the probes with the “T” allele of rs6486433 and the “A” allele of rs3763869 yielded additional shift bands by contrast to the “G” allele of rs6486433 and “G” allele of rs3763869, respectively. This suggests that rs6486433 and rs3763869 in the *WEE1* promoter region can mediate allele-specific transcriptional factor binding *in vitro*, which is the molecular basis for the differential transcriptional strength of *WEE1* promoter haplotypes (Fig. 4D).

## Discussion

DNA-damaging agents are the most commonly used anti-cancer drugs for the treatment of NSCLC patients. In cancer cells, DNA damage and repair pathway activation plays a critical role in the sensitivity of DNA-damaging agents^18^. Numerous studies have revealed that DNA damage response pathways can activate cell cycle checkpoints to arrest the cell either temporarily or permanently (senescence). It is well established that p53 is responsible for G1 checkpoint regulation^19^ and that *WEE1* tyrosine kinase is the major regulator for the G2 checkpoint^20^. The majority of tumors in NSCLC patients are found to be p53-mutated or p53-deficient and rely only on the *WEE1* G_2_ checkpoint for cell cycle arrest after treatment with DNA-damaging agents. Although *WEE1* somatic variation is seldom present in NSCLC, we find that germline variation in *WEE1* has great impact on the efficacy of DNA-damaging agents in the treatment of advanced NSCLC.

DNA-damaging drugs will induce G_1_- or G_2_-arrest for the DNA repair process, which is a major determinant of the sensitivity towards cisplatin^21^. Abrogation of cell cycle arrest will force cells into mitosis with unrepaired DNA and eventually lead to apoptosis or mitotic catastrophe^22^,^23^. Nurse *et al*. first demonstrated that *WEE1* is involved in the regulation of G_2_ checkpoints^24^ while Rowley *et al*. found that *Schizosaccharomyces pombe* strains with defective or abolished *WEE1* function failed to arrest cells in G_2_ following treatment with cisplatin and were hypersensitive to cisplatin treatment^25^. Expression of *WEE1* delays the mitotic entry of cells with radiation-induced DNA damage and the lengthening of the G_2_ phase depends on the expression of *WEE1*^26^. Inhibition of *WEE1* by siRNA, small molecule inhibitors, or other proteins exposed human cancer cells to DNA-damaging agents, resulting in abrogation of the G_2_ arrest, premature termination of DNA repair, and cell death, leading to increased sensitivity towards DNA-damaging agents in different types of carcinomas^27^,^28^,^29^. To the best of our knowledge, the current study demonstrates for the first time that *WEE1* kinase rs3910384 “G/G” allele is strongly associated with the therapeutic efficacy of platinum-gemcitabine doublet regimen, consisting of both DNA-damaging agents.

According to the NCCN (National Comprehensive Cancer Network) guidelines, platinum-based doublet chemotherapy is the most common first-line treatment for patients with advanced NSCLC^30^. At present, choosing a drug combination for NSCLC chemotherapy is equivocal because all of the combinations provide almost similar efficacy; a physician’s selection of a particular chemotherapy regimen depends on the patient’s tolerance for the adverse side effects, not the efficacy of the regimen. Platinum-based chemotherapy provides a slightly longer OS of only 1.5 months (from 4.5 months to 6 months) and a 9% absolute increase of 1-year-survival (from 20% to 29%) than the best supportive care in unselected NSCLC patients^31^. Our data indicate that *WEE1* rs3910384 G/G can be used as a predictive biomarker for platinum-gemcitabine regimen with the benefit of 13.5 months longer median OS, 144% relative increase in 1-year survival rate (from 22.1% to 54.3%), 163% relative increase in 2-year survival rate (from 14.7% to 38.8%), and 274% relative increase in 3-year survival rate (from 7.4% to 27.7%) following treatment with platinum-gemcitabine regimen. The validation in the other 264 patients also shows that *WEE1* rs3910384 is significantly associated with the efficacy of platinum-gemcitabine regimen. *WEE1* rs3910384 G/G genotype accounts for 33.6% in Chinese, 13.4% in Western, and 17.5% in African patients (Table S3). We postulate that *WEE1* rs3910384 G/G is a potential biomarker for indicating the platinum-gemcitabine regimen in Chinese NSCLC patients. Whether it can be used as a biomarker in other populations needs to be further validated.

Among the four *WEE1* tag SNPs we examined, rs3910384 is near the *WEE1* core promoter. Although the direct function of rs3910384 is still unknown, linkage disequilibrium analysis demonstrated that rs3910384 was closely linked with the 3 predicted regulatory SNPs rs6486433 (D’ = 0.91), rs3763868 (D’ = 0.88) and rs3763869 (D’ = 0.89) within the transcription-factor-binding sites of the *WEE1* promoter. The MAF of rs6486433 (MAF = 0.462), rs3763868 (MAF = 0.499), and rs3763869 (MAF = 0.493) are also similar to rs3910384 (0.486) in the HapMap population. Nearly ninety percent of the patients harbor one of the two major haplotypes of the *WEE1* promoter, *i.e*. rs3910384 “G” allele-linked T/G/G (rs6486433/rs3763869/rs3763868, 43.4%) or rs3910384 “A” allele-linked G/A/C (44.8%). After cloning the two major *WEE1* haplotype promoters into PGL4 luciferase reporter plasmids, we confirmed the allele-specific transcription regulation of *WEE1* expression in A549 and H1299 cells after treatment with DNA-damaging chemotherapy agents. The *WEE1* haplotype promoter with rs3910384 “A” allele-linked G/A/C can drive much higher RLU than the counterpart with rs3910384 “G” allele-linked T/G/G after DNA damage induced by cisplatin, gemcitabine, or a combination. According to SNPINFO website (http://snpinfo.niehs.nih.gov/snpinfo/snpfunc.htm), the *WEE1* promoter allele-specific TFBS (transcription factor binding sites) include C/EBP, ETS, or SPZ1 for rs3763869, MAZ for rs3763868, and C/EBP, ETS, GCNF, or HNF4α for rs6486433. To validate the allele-specific transcription factor binding in the *WEE1* promoter, we performed EMSA using nuclear extracts from lung cancer cell lines H1299 and A549, and found that rs6486433 and rs3763869, but not rs3763868, can mediate allele-specific transcription factor binding *in vitro*. The exact mechanism of the *WEE1* promoter allele-specific transcription factors and its regulation of *WEE1* expression needs to be further studied in detail.

As reported, Wee1 kinase is a gatekeeper molecule and regulates the G_2_-cell-cycle checkpoint arrest for pre-mitotic DNA repair^27^. Wee1-induced G_2_-arrest plays a critical role in sensitivity to DNA-damaging agents or radiation^12^,^32^. The Wee1 concentration in human cells is highly regulated during the cell cycle. Wee1 total protein (mRNA) is synthesized *de novo* at S and G_2_ phases, then phosphorylated, ubiquitinated, and eliminated through proteasome-dependent degradation during M phase^33^,^34^,^35^. The activity of Wee1 increases during S and G_2_ phases in parallel with the increase in the protein level^36^. Wee1 protein is significantly increased and induces G_2_ arrest following DNA damage by drugs or radiation^11^. Although Wee1 expression can be regulated in many ways, the *de novo* synthesis of Wee1 is the major event that occurs as a response to DNA damage. The discrepancy of the strength of the *WEE1* promoter haplotypes determines the level of Wee1 expression in the DNA damage response. This SNP allele-specific transcription of Wee1 provides the molecular explanation for the association of rs3910384 “G” allele with the increased sensitivity to DNA-damaging drugs.

Several efficacy-predicting biomarkers for chemotherapy in lung cancer patients have been reported. *ERCC1* for platinum^37^,^38^, *RRM1* for gemcitabine^39^,^40^, and *TUBB3* for paclitaxel^41^ have been investigated in preclinical and/or in clinical studies involving small numbers of patients; however, their predictive roles are still debated. Crucially, these biomarkers cannot be validated in other studies or in multicenter clinical trials^42^,^43^,^44^, possibly due to either the instability of the mRNA or protein expression or unreproducible quantitative methods. Detection of germline or somatic mutations can provide highly reproducible results and is more suitable as a biomarker in clinical practice. *WEE1* rs3910384 G/G genotype detection can be a valuable biomarker for the platinum-gemcitabine chemotherapy regimen.

Currently, *WEE1* inhibitors MK-1775 and PD-166285 are being tested in preclinical and clinical studies of human carcinoma^14^,^45^. Synergy between *WEE1* inhibitors (MK-1775 or PD-166285) and DNA-damaging agents has been reported^14^,^46^,^47^. MK-1775 achieved favorable phase I pharmacokinetic and pharmacodynamic endpoints in combination with carboplatin, cisplatin, and gemcitabine and is under further investigation as a chemosensitizer in a phase II trial^48^. Our finding suggests that a population of NSCLC patients with *WEE1* rs3910384 G/G genotype is much more sensitive to the platinum-gemcitabine chemotherapy regimen. This novel biomarker, the *WEE1* rs3910384 G/G genotype, can provide 13.5 months longer overall survival and 374% relative increase (from 7.4% to 27.7%) of the 3-year-survival rate for advanced NSCLC patients treated with platinum-gemcitabine chemotherapy regimen. As such, it is a potential tool for deciding on combination chemotherapy options to facilitate personalized chemotherapy for NSCLC. However, this conclusion was derived from unselected NSCLC patients regardless of somatic variation of EGFR and ALK. For NSCLC patients without EGFR mutation or ALK rearrangement, platinum-based chemotherapy is still the first-line option of treatment. Determining whether the *WEE1* rs3910384 G/G genotype can be used a biomarker for the option of platinum-gemcitabine regimen in NSCLC patients harboring no EGFR mutation and ALK rearrangement will better define this biomarker for the platinum-gemcitabine regimen and it is worth further validation in the future.

## Additional Information

**How to cite this article**: Liu, D. *et al*. WEE1 kinase polymorphism as a predictive biomarker for efficacy of platinum-gemcitabine doublet chemotherapy in advanced non-small cell lung cancer patients. *Sci. Rep*. **5**, 11114; doi: 10.1038/srep11114 (2015).

## Supplementary Material

Supplementary Information

## Figures and Tables

**Table 1:** Univariate Cox’s regression analysis of prognostic factors for overall survival in the 663 advanced NSCLC patients treated with platinum-based chemotherapy.

Variables	N = 663	mOS (95%CI)	HR (95% CI)	*p*
Median age, y (range)	58(26–80)			0.107
<60	333(50.2%)	19.4(17.4–21.3)	R	
≥60	329(49.6%)	16.8(14.8–18.8)	1.16(0.97–1.39)	
Gender				0.032^*^
Male	465 (70.1%)	17.2(15.4–19.0)	R	
Female	198 (29.9%)	20.0(17.2–22.8)	0.80(0.65–0.98)	
Smoking history				0.026^*^
Nonsmoker	277 (41.8%)	19.7(17.2–22.1)	R	
Ever Smoker	386 (58.2%)	16.9(15.0–18.9)	1.24(1.03–1.49)	
ECOG PS				0.088
1	606(91.4%)	18.6(17.1–20.1)	R	
2	54(8.1%)	17.7(10.6–24.8)	1.32(0.96–1.81)	
Tumor histology				0.016^*^
Adenocarcinoma	430 (64.9%)	19.1(17.3–20.8)	R	
Squamous cell	141 (21.3%)	15.0(11.9–18.1)	1.34(1.08–1.67)	0.009
Adenosquamous cell	13 (2.0%)	12.7(5.6–19.8)	1.73(0.95–3.16)	0.075
Other NSCLC^a^	79 (11.9%)	17.4(12.8–21.9)	1.27(0.96–1.68)	0.097
Clinical stage				0.048^*^
IIIa	49 (7.4%)	27.0(17.9–36.1)	1.16(1.00–1.34)	
IIIb	189 (28.5%)	18.4(16.4–20.4)		
IV	423 (63.8%)	17.7(15.5–19.8)		
Doublet regimen				0.992
Platinum-gemcitabine	152(22.9%)	17.8(15.3–20.4)	R	
Platinum-tubulin-targeting drugs^b^	472(71.2%)	18.6(16.6–20.5)	1.00(0.81–1.25)	0.971
Other combination^c^	39(5.9%)	18.4(11.6–25.2)	1.03(0.69–1.53)	0.903
rs3829254				0.07
A/A	435(65.6%)	19.1(17.1–21.0)	R	
A/G	210(31.7%)	16.2(13.8–18.5)	1.24(1.03–1.51)	0.027*
G/G	16(2.4%)	11.7(1.3–22.1)	1.32(0.72–2.42)	0.363
dominant				
AA +AG	645(98%)	18.6(17.1–20.0)	1.23(0.08–2.24)	0.498
GG	16(2%)	11.7(1.3–22.1)		
recessive				
AA	435(65.8%)	19.1(17.1–21.0)	1.25(1.03–1.51)	0.022*
GG+AG	226(34.2%)	16.2(13.9–18.4)		
additive				
NA	NA	NA	1.22(1.03–1.44)	0.023*
rs3910384				0.022*
G/G	268(40.4%)	20.4(17.6–23.2)	R	
A/G	298(44.9%)	16.9(14.9–19.0)	1.29(1.05–1.57)	0.014*
A/A	89(13.4%)	16.2(12.1–20.3)	1.36(1.03–1.80)	0.033*
dominant				
AA+AG	387(59.1%)	16.9(15.1–18.7)	0.77(0.64–0.93)	0.006*
GG	268(40.9%)	20.4(17.6–23.2)		
recessive				
AA	89(13.6%)	16.2(12.1–20.3)	0.84(0.65–1.09)	0.187
GG+AG	566(86.4%)	18.7(17.1–20.3)		
additive				
NA	NA	NA	0.84(0.74–0.96)	0.009*
rs4370932				0.088
A/A	164(24.7%)	19.4(16.1–22.6)	1	
A/G	317(47.8%)	18.4(16.1–20.8)	1.14(0.9–1.43)	0.284
G/G	182(27.5%)	16.1(13.5–18.8)	1.32(1.03–1.7)	0.029*
dominant				
AA+AG	481(72.5%)	19.0(17.2–20.8)	1.22(1.00–1.48)	0.052
GG	182(27.5%)	16.1(13.5–18.8)		
recessive				
AA	164(24.7%)	19.4(16.1–22.6)	1.20(0.97–1.49)	0.095
GG+AG	499(75.3%)	17.5(15.9–19.2)		
additive				
NA	NA	NA	1.15(1.02–1.30)	0.028*
rs1049403				0.159
A/A	433(65.3%)	18.9(16.9–20.9)	1	
A/G	206(31.1%)	17.0(14.6–19.5)	1.21(1.0–1.47)	0.056
G/G	23(3.5%)	16.9(11.3–22.6)	1.1(0.66–1.85)	0.716
dominant				
AA+AG	639(96.5%)	18.4(17.0–19.9)	1.03(0.62–1.73)	0.897
GG	23(3.5%)	16.9(11.3–22.6)		
recessive				
AA	433(65.4%)	18.9(16.9–20.9)	1.20(0.99–1.45)	0.060
GG+AG	229(34.6%)	17.0(14.7–19.3)		
additive				
NA	NA	NA	1.15(0.98–1.35)	0.094

^*^p < 0.05. Abbrev.: mOS (months): median overall survival, HR: hazard ratio, NA: Not available, R: Reference.

**Table 2:** Multivariate Cox’s regression analysis of prognostic factors for overall survival in the 663 advanced NSCLC patients treated with platinum-based chemotherapy.

Variables	HR (95% CI)	*p*
Gender (F *vs*. M)	0.87(0.70–1.08)	0.199
Smoking (ever *vs*. never)	1.07(0.81–1.40)	0.644
Clinical stage (IV *vs*. IIIb *vs*. IIIa)	1.22(1.05–1.42)	8.0E-3*
Histology		4.3E-4*
adenocarcinoma	R	
squamous	1.42(1.13–1.79)	2.4E-3*
adenosquamous	2.74(1.45–5.15)	1.9E-3*
others	1.30(0.98–1.74)	0.073
rs3829254		
recessive	1.17(0.93–1.48)	0.178
rs3910384		
dominant	0.76(0.63–0.91)	4.0E-3*
rs4370932		
additive	1.01(0.85–1.21)	0.917

*p < 0.05. All the variables yielding *P*-values < 0.05 in the univariate analysis were used for multivariate Cox’s regression: Gender, smoking, clinical stage, histology, the rs3910384 dominant model, the rs3829254 recessive model, and the rs4370932 additive model.

**Table 3:** Cox’s regression analysis of prognostic factors for overall survival in the 152 NSCLC patients treated with platinum-gemcitabine regimen.

		Univariate			Multivariate	
mOS(95%CI)		HR (95% CI)	*p*		HR (95% CI)	*p*
Age						
<60	19.5(16.3–22.8)					
≥60	16.1(12.3–20.0)	1.36(0.92–2.01)	0.125		1.34(0.90–2.01)	0.155
Gender:						
Male	15.5(12.2–18.8)					
Female	20.0(15.3–24.7)	0.73(0.49–1.08)	0.118		0.81(0.51–1.27)	0.351
Smoking history:						
Never smoker	19.0(15.6–22.5)					
Ever Smoker	16.1(11.9–20.4)	1.07(0.73–1.58)	0.724		0.75(0.42–1.32)	0.317
ECOG PS:						
1	18.4(15.4–21.4)					
2	17.5(14.4–20.6)	1.68(0.85–3.34)	0.139		1.19(0.57–2.49)	0.641
Clinical stage						
IIIa	19.0					
IIIb	16.8(12.2–21.4)	1.18(0.87–1.61)	0.294		1.27(0.90–1.79)	0.174
IV	17.8(14.6–21.1)					
Histology			0.05*			0.037*
adenocarcinoma	19.5(16.1–23.0)	1			R	
squamous cell	13.7(9.4–18.1)	1.50(0.92–2.45)	0.107		1.60(0.96–2.65)	0.069
adenosquamous	12.7(0–30.1)	3.98(1.23–12.87)	0.021		4.11(1.27–13.31)	0.018*
others	18.8(12.3–25.3)	1.50(0.77–2.91)	0.235		1.49(0.74–2.99)	0.262
rs3829254	recessive					
A/A	19.5(14.4–24.7)					
AG+GG	14.7(10.7–18.7)	1.61(1.09–2.38)	0.018*		1.15(0.67–1.98)	0.603
rs3910384	dominant					
G/G	28.2(20.5–35.9)	0.43(0.28–0.65)	7.6E-5*		0.42(0.27–0.64)	6.9E-5*
AA+AG	14.7(12.8–16.6)					
rs4370932	additive					
	NA	NA	0.012*		1.04(0.51–2.13)	0.917
rs1049403	recessive					
A/A	19.5(14.4–24.7)					
AG+GG	14.4(12.6–16.2)	1.62(1.10–2.39)	0.015*		0.82(0.27–2.50)	0.726

*p < 0.05. Abbrev.: mOS (months): median overall survival, HR: hazard ratio, NA: Not available, R: Reference. Variables used in the multivariate analysis: Age, Gender, smoking history, clinical stage, histology, the rs3829254 recessive model, the rs3910384 dominant model, the rs4370932 additive model and the rs1049403 recessive model.

**Table 4:** Association of *WEE1* rs3910384 dominate model with the disease control rate in patients treated platinum-gemcitabine regimen.

Genotype	ORR(CR+PR)				DCR (CR+PR+SD)		
	**%**	**X**^**2**^	***p***		**%**	**X**^**2**^	***p***
A/A+A/G	13.8%(12/87)	0.432	0.511		72.4%(63/87)	3.587	0.058
G/G	17.7%(11/62)				85.5%(53/62)		

Abbrev: OCR: objective response rate; DCR: disease control rate; CR: complete response; PR: partial response; SD: stable disease.

## References

[b1] Jemal A. . Global cancer statistics. CA Cancer J Clin 61, 69–90 (2011). 21296855 10.3322/caac.20107

[b2] Chen W. . Report of incidence and mortality in China cancer registries, 2009. Chin J Cancer Res 25, 10–21 (2013). 23372337 10.3978/j.issn.1000-9604.2012.12.04 PMC3555299

[b3] Ferlay J. . Estimates of worldwide burden of cancer in 2008: GLOBOCAN 2008. Int J Cancer 127, 2893–2917 (2010). 21351269 10.1002/ijc.25516

[b4] Janku F., Stewart D. J. & Kurzrock R. Targeted therapy in non-small-cell lung cancer–is it becoming a reality? Nat Rev Clin Oncol 7, 401–414 (2010). 20551945 10.1038/nrclinonc.2010.64

[b5] Ma P. C. Personalized targeted therapy in advanced non-small cell lung cancer. Cleve Clin J Med 79 Electronic Suppl 1, eS56–60 (2012). 22614968 10.3949/ccjm.79.s2.12

[b6] Ozkaya S., Findik S., Dirican A. & Atici A. G. Long-term survival rates of patients with stage IIIB and IV non-small cell lung cancer treated with cisplatin plus vinorelbine or gemcitabine. Exp Ther Med 4, 1035–1038 (2012). 23226770 10.3892/etm.2012.714 PMC3494120

[b7] Boulikas T. & Vougiouka M. Cisplatin and platinum drugs at the molecular level. (Review). Oncol Rep 10, 1663–1682 (2003). 14534679

[b8] Plunkett W. . Gemcitabine: metabolism, mechanisms of action, and self-potentiation. Semin Oncol 22, 3–10 (1995). 7481842

[b9] Schiller J. H. . Comparison of four chemotherapy regimens for advanced non-small-cell lung cancer. N Engl J Med 346, 92–98 (2002). 11784875 10.1056/NEJMoa011954

[b10] McGowan C. H. & Russell P. Cell cycle regulation of human *WEE1*. EMBO J 14, 2166–2175 (1995). 7774574 10.1002/j.1460-2075.1995.tb07210.x PMC398322

[b11] Raleigh J. M. & O’Connell M. J. The G(2) DNA damage checkpoint targets both Wee1 and Cdc25. J Cell Sci 113 (**Pt 10**) 1727–1736 (2000). 10769204 10.1242/jcs.113.10.1727

[b12] Wang Y., Decker S. J. & Sebolt-Leopold J. Knockdown of Chk1, Wee1 and Myt1 by RNA interference abrogates G2 checkpoint and induces apoptosis. Cancer Biol Ther 3, 305–313 (2004). 14726685 10.4161/cbt.3.3.697

[b13] PosthumaDeBoer J. . *WEE1* inhibition sensitizes osteosarcoma to radiotherapy. BMC Cancer 11, 156 (2011). 21529352 10.1186/1471-2407-11-156 PMC3103478

[b14] De Witt Hamer P. C., Mir S. E., Noske D., Van Noorden C. J. & Wurdinger T. *WEE1* kinase targeting combined with DNA-damaging cancer therapy catalyzes mitotic catastrophe. Clin Cancer Res 17, 4200–4207 (2011). 21562035 10.1158/1078-0432.CCR-10-2537

[b15] Edmead C., Kanthou C. & Benzakour O. Thrombin activates transcription factors sp1, NF-kappaB, and CREB: importance of the use of phosphatase inhibitors during nuclear protein extraction for the assessment of transcription factor DNA-binding activities. Anal Biochem 275, 180–186 (1999). 10552902 10.1006/abio.1999.4313

[b16] Hirai H. . Small-molecule inhibition of Wee1 kinase by MK-1775 selectively sensitizes p53-deficient tumor cells to DNA-damaging agents. Mol Cancer Ther 8, 2992–3000 (2009). 19887545 10.1158/1535-7163.MCT-09-0463

[b17] Stathis A. & Oza A. Targeting Wee1-like protein kinase to treat cancer. Drug News Perspect 23, 425–429 (2010). 20862394 10.1358/dnp.2010.23.7.1490760

[b18] Cheung-Ong K., Giaever G. & Nislow C. DNA-damaging agents in cancer chemotherapy: serendipity and chemical biology. Chem Biol 20, 648–659 (2013). 23706631 10.1016/j.chembiol.2013.04.007

[b19] el-Deiry W. S. . WAF1/CIP1 is induced in p53-mediated G1 arrest and apoptosis. Cancer Res 54, 1169–1174 (1994). 8118801

[b20] Masuda H., Fong C. S., Ohtsuki C., Haraguchi T. & Hiraoka Y. Spatiotemporal regulations of Wee1 at the G2/M transition. Mol Biol Cell 22, 555–569 (2011). 21233285 10.1091/mbc.E10-07-0644 PMC3046054

[b21] Brozovic A. . Cisplatin sensitivity is related to late DNA damage processing and checkpoint control rather than to the early DNA damage response. Mutat Res 670, 32–41 (2009). 19616017 10.1016/j.mrfmmm.2009.07.002

[b22] Leijen S., Beijnen J. H. & Schellens J. H. Abrogation of the G2 checkpoint by inhibition of Wee-1 kinase results in sensitization of p53-deficient tumor cells to DNA-damaging agents. Curr Clin Pharmacol 5, 186–191 (2010). 20406171 10.2174/157488410791498824

[b23] Al-Ejeh F. . Harnessing the complexity of DNA-damage response pathways to improve cancer treatment outcomes. Oncogene 29, 6085–6098 (2010). 20818418 10.1038/onc.2010.407

[b24] Russell P. & Nurse P. Negative regulation of mitosis by wee1+, a gene encoding a protein kinase homolog. Cell 49, 559–567 (1987). 3032459 10.1016/0092-8674(87)90458-2

[b25] Thiebaut F., Enns R. & Howell S. B. Cisplatin sensitivity correlates with its ability to cause cell cycle arrest via a wee1 kinase-dependent pathway in Schizosaccharomyces pombe. J Cell Physiol 159, 506–514 (1994). 8188765 10.1002/jcp.1041590315

[b26] Rowley R., Hudson J. & Young P. G. The wee1 protein kinase is required for radiation-induced mitotic delay. Nature 356, 353–355 (1992). 1549179 10.1038/356353a0

[b27] Mir S. E. . In silico analysis of kinase expression identifies *WEE1* as a gatekeeper against mitotic catastrophe in glioblastoma. Cancer Cell 18, 244–257 (2010). 20832752 10.1016/j.ccr.2010.08.011 PMC3115571

[b28] Pouliot L. M. . Cisplatin sensitivity mediated by *WEE1* and CHK1 is mediated by miR-155 and the miR-15 family. Cancer Res 72, 5945–5955 (2012). 22942255 10.1158/0008-5472.CAN-12-1400 PMC3500396

[b29] Wang F. . Transcriptional repression of *WEE1* by Kruppel-like factor 2 is involved in DNA damage-induced apoptosis. Oncogene 24, 3875–3885 (2005). 15735666 10.1038/sj.onc.1208546

[b30] Ettinger D. S. . Non-small cell lung cancer, version 2.2013. J Natl Compr Canc Netw 11, 645–653; quiz 653 (2013). 23744864 10.6004/jnccn.2013.0084

[b31] Non-Small Cell Lung Cancer Collaborative, G. Chemotherapy and supportive care versus supportive care alone for advanced non-small cell lung cancer. *Cochrane Database Syst Rev* **12**, CD007309,doi: 007310.001002/14651858.CD14007309.pub14651852. (2010). 10.1002/14651858.CD007309.pub2 PMC11380090 20464750

[b32] Sarcar B. . Targeting radiation-induced G(2) checkpoint activation with the Wee-1 inhibitor MK-1775 in glioblastoma cell lines. Mol Cancer Ther 10, 2405–2414 (2011). 21992793 10.1158/1535-7163.MCT-11-0469 PMC5753756

[b33] O’Connell M. J., Raleigh J. M., Verkade H. M. & Nurse P. Chk1 is a wee1 kinase in the G2 DNA damage checkpoint inhibiting cdc2 by Y15 phosphorylation. The EMBO journal 16, 545–554 (1997). 9034337 10.1093/emboj/16.3.545 PMC1169658

[b34] Watanabe N. . M-phase kinases induce phospho-dependent ubiquitination of somatic Wee1 by SCFbeta-TrCP. Proc Natl Acad Sci USA 101, 4419–4424 (2004). 15070733 10.1073/pnas.0307700101 PMC384762

[b35] Dai Y. & Grant S. New insights into checkpoint kinase 1 in the DNA damage response signaling network. Clin Cancer Res 16, 376–383 (2010). 20068082 10.1158/1078-0432.CCR-09-1029 PMC2939735

[b36] Watanabe N., Broome M. & Hunter T. Regulation of the human *WEE1*Hu CDK tyrosine 15-kinase during the cell cycle. EMBO J 14, 1878–1891 (1995). 7743995 10.1002/j.1460-2075.1995.tb07180.x PMC398287

[b37] Lord R. V. . Low ERCC1 expression correlates with prolonged survival after cisplatin plus gemcitabine chemotherapy in non-small cell lung cancer. Clin Cancer Res 8, 2286–2291 (2002). 12114432

[b38] Tantraworasin A. . The prognostic value of ERCC1 and RRM1 gene expression in completely resected non-small cell lung cancer: tumor recurrence and overall survival. Cancer Manag Res 5, 327–336 (2013). 24124391 10.2147/CMAR.S52073 PMC3794893

[b39] Olaussen K. A. . DNA repair by ERCC1 in non-small-cell lung cancer and cisplatin-based adjuvant chemotherapy. N Engl J Med 355, 983–991 (2006). 16957145 10.1056/NEJMoa060570

[b40] Zheng Z. . DNA synthesis and repair genes RRM1 and ERCC1 in lung cancer. N Engl J Med 356, 800–808 (2007). 17314339 10.1056/NEJMoa065411

[b41] Zhang H. L. . Association between class III beta-tubulin expression and response to paclitaxel/vinorebine-based chemotherapy for non-small cell lung cancer: a meta-analysis. Lung Cancer 77, 9–15 (2012). 22306125 10.1016/j.lungcan.2012.01.005

[b42] Reiman T. . Cross-validation study of class III beta-tubulin as a predictive marker for benefit from adjuvant chemotherapy in resected non-small-cell lung cancer: analysis of four randomized trials. Ann Oncol 23, 86–93 (2012). 21471564 10.1093/annonc/mdr033 PMC3276322

[b43] Yamashita F. . Prognostic value of EGFR mutation and ERCC1 in patients with non-small cell lung cancer undergoing platinum-based chemotherapy. PLoS One 8, e71356 (2013). 23940741 10.1371/journal.pone.0071356 PMC3734014

[b44] Bepler G. . Randomized international phase III trial of ERCC1 and RRM1 expression-based chemotherapy versus gemcitabine/carboplatin in advanced non-small-cell lung cancer. J Clin Oncol 31, 2404–2412 (2013). 23690416 10.1200/JCO.2012.46.9783 PMC3691357

[b45] Guertin A. D. . Preclinical evaluation of the *WEE1* inhibitor MK-1775 as single-agent anticancer therapy. Mol Cancer Ther 12, 1442–1452 (2013). 23699655 10.1158/1535-7163.MCT-13-0025

[b46] Rajeshkumar N. V. . MK-1775, a potent Wee1 inhibitor, synergizes with gemcitabine to achieve tumor regressions, selectively in p53-deficient pancreatic cancer xenografts. Clin Cancer Res 17, 2799–2806 (2011). 21389100 10.1158/1078-0432.CCR-10-2580 PMC3307341

[b47] Indovina P. . Abrogating G(2)/M checkpoint through *WEE1* inhibition in combination with chemotherapy as a promising therapeutic approach for mesothelioma. Cancer Biol Ther 15, 380–388 (2014). 24365782 10.4161/cbt.27623 PMC3979815

[b48] Guertin A. D. . Unique functions of CHK1 and *WEE1* underlie synergistic anti-tumor activity upon pharmacologic inhibition. Cancer Cell Int 12, 45 (2012). 23148684 10.1186/1475-2867-12-45 PMC3517755

