# Supplementary material for: WEE1 kinase polymorphism as a predictive biomarker for efficacy of platinum-gemcitabine doublet chemotherapy in advanced non-small cell lung cancer patients
Source: Sci Rep. 2015 Jun 9;5:11114. doi: 10.1038/srep11114 (PMC4460872; doi:10.1038/srep11114)
Supplement: Supplementary Information [file srep11114-s1.doc]

***WEE1* kinase polymorphism as a predictive biomarker for** **efficacy of platinum-gemcitabine doublet chemotherapy in advanced non-small cell lung cancer patients**

Di Liu^1*^, Chunyan Wu^2*^, Yuli Jiao^1^, Likun Hou^2^, Daru Lu^4^, Hui Zheng^3^, Chang Chen^3^, Ji Qian^4^ , Ke Fei^3^ , Bo Su^1^

^1^ Central Laboratory, ^2^ Department of Pathology, ^3^ Department of Thoracic Surgery, Shanghai Pulmonary Hospital, Tongji University School of Medicine

^4^ Fudan University Shanghai Cancer Center, State Key Laboratory of Genetic Engineering and MOE Key Laboratory of Contemporary Anthropology, School of Life Sciences, Fudan University, Shanghai, P.R. China

*These authors contributed equally to this article.

Authors for correspondence: Bo Su, PhD, Central Laboratory ([su_bo_s@hotmail.com](mailto:su_bo_s@hotmail.com)); Ke Fei, MD, Department of Thoracic Surgery ([ffeik@126.com](mailto:ffeik@126.com)); Shanghai Pulmonary Hospital, Tongji University School of Medicine, No. 507, Zheng Min Road, Shanghai, 200433, P.R. China.

Phone: +86-21-65115006

Fax: +86-21-65111298

**Supplementary Table S1. The 5’-biotin-labeled double-stranded oligonucleotides probes of the 3 rs3910384 –linked SNPs in WEE1 promoter region for EMSA.**

| SNPs | Genotype | sequence |
| --- | --- | --- |
| rs6486433 | reference | 5'-Biotin-CCCGAGGCCCGAGTCACCATTTCC  GGCTCAGACCTCGACCCTGAACGTGGCTGCCCACT-3' |
|  | allele | 5'-Biotin-CCCGAGGCCCGAGTCACCATTTCC  GGCTCAGACCTCGACCCGGAACGTGGCTGCCCACT-3' |
| rs3763869 | reference | 5'-Biotin-CCCAGCTCGCAGAGACCCGGAGG  CGCTGCCCGGCCGCCTGCCCC-3' |
|  | allele | 5'-Biotin-CCCAGCTCGCAGAGACCCGGAAG  CGCTGCCCGGCCGCCTGCCCC-3' |
| rs3763868 | reference | 5'-Biotin-GGAGGAGCAGCGAGGGGGGTGCG  TCCAGGCCGGCTTTCGGG-3' |
|  | allele | 5'-Biotin-GGAGGAGCAGCGAGGGGGCTGCG  TCCAGGCCGGCTTTCGGG-3' |

Underlined letters represent the SNP alleles.

**Supplementary Table S2. Clinical characteristics of the NSCLC patients in the test cohort.**

| Patients’ characteristics | N = 663 |
| --- | --- |
| Median age, y (range) | 58(26-80) |
| <60 | 333(50.2%) |
| ≥60 | 329(49.6%) |
| Gender |  |
| Male | 465 (70.1%) |
| Female | 198 (29.9%) |
| Smoking history |  |
| Ever Smoker | 386 (58.2%) |
| Nonsmoker | 277 (41.8%) |
| ECOG PS |  |
| 1 | 606(91.4%) |
| 2 | 54(8.1%) |
| Tumor histology |  |
| Adenocarcinoma | 430 (64.9%) |
| Squamous cell | 141 (21.3%) |
| Adenosquamous cell | 13 (2.0%) |
| Other NSCLC^a^ | 79 (11.9%) |
| Clinical stage |  |
| IIIa | 49 (7.4%) |
| IIIb | 189 (28.5%) |
| IV | 423 (63.8%) |
| Duplet regimens |  |
| Platinum-gemcitabine | 152(22.9%) |
| Platinum-tubulin-targeting drugs^b^ | 472(71.2%) |
| Other combination^c^ | 39(5.9%) |

^a^ Others NSCLC included mixed-cell, neuroendocrine carcinoma, or undifferentiated carcinoma.

^b^ Tubulin-targeting drugs includes paclitaxel (n=203), docetaxel (n=54) or navelbine (n=215).

^c^Other combination included etoposide or bevacizumab.

**p*<0.05, Log-Rank test.

Abbreviation: mOS, midian overall survival; ECOG, Eastern Cooperative Oncology Group; PS, performance status.

**Supplementary Table S3. Genotype frequencies of WEE1 tag SNPs in current data, or in CHB, CEU and YRI from Hapmap SNP database.**

| tag SNPs | Location | Allele frequencies | | | |  |
| --- | --- | --- | --- | --- | --- | --- |
|  |  | Current Data | CHB | CEU | YRI | *p* |
| rs3829254 | 5'near gene | | |  |  |  |
| MAF |  | 0.183 (G) | 0.242 (G) | 0.208 (G) | 0.339(G) | <0.001 |
| A/A |  | 435(65.8%) | 34(57.5%) | 38(62.7%) | 52(44.1%) | |
| A/G |  | 210(31.8%) | 22(36.7%) | 19(31.7%) | 52(44.1%) | |
| G/G |  | 16(2.4%) | 4(5.9%) | 3(4.3%) | 14(11.8%) | |
| rs3910384 | intron | |  |  |  |  |
| MAF |  | 0.363 (A) | 0.401 (A) | 0.411 (G) | 0.417(A) | <0.001 |
| A/A |  | 89(13.6%) | 19(13.9%) | 35(31.2%) | 26(18.1%) | |
| A/G |  | 298(45.5%) | 72(52.6%) | 62(55.4%) | 68(47.2%) | |
| G/G |  | 268(40.9%) | 46(33.6%) | 15(13.4%) | 50(34.7%) | |
| rs4370932 | intron | |  |  |  |  |
| MAF |  | 0.486 (A) | 0.459 (A) | 0.376 (A) | 0.413(G) | <0.001 |
| A/A |  | 164(24.7%) | 25(18.8%) | 14(12.4%) | 50(34.5%) | |
| A/G |  | 317(47.8%) | 72(54.1%) | 57(50.4%) | 65(44.8%) | |
| G/G |  | 182(27.5%) | 36(27.1%) | 42(37.2%) | 30(20.7%) | |
| rs1049403 | 3' UTR | |  |  |  |  |
| MAF |  | 0.190 (G) | 0.197 (G) | 0.143 (G) | 0.061(G) | <0.001 |
| A/A |  | 433(65.4%) | 89(65%) | 82(73.2%) | 129(87.8%) | |
| A/G |  | 206(31.1%) | 42(30.7%) | 28(25%) | 18(12.2%) | |
| G/G |  | 23(3.5%) | 6(4.4%) | 2(1.8%) | 0 |  |

Abbreviation: UTR, untranslated region; MAF, Minor allele frequency; CHB, Han Chinese in Beijing, China ;CEU, Utah residents with Northern and Western European ancestry from the CEPH collection; YRI, Yoruban in Ibadan, Nigeria.

**Supplementary** **Table S4.** Univariate and multivariate Cox's regression analysis of prognostic factors for progression free survival in the 663 patients with NSCLC.

|  | mPFS (95%CI) | Univariate | |  | Multivariate | |
| --- | --- | --- | --- | --- | --- | --- |
|  |  | HR (95% CI) | *p* |  | HR (95% CI) | *p* |
| Age |  |  |  |  |  |  |
| ＜60 | 5.9(5.0-6.8) |  |  |  |  |  |
| ≥60 | 7.1(5.7-8.5) | 0.93(0.77-1.12) | 0.456 |  | 0.93(0.77-1.13) | 0.461 |
| Gender: |  |  |  |  |  |  |
| Male | 6.8(5.9-7.7) |  |  |  |  |  |
| Female | 5.8(4.5-7.0) | 1.16(0.95-1.42) | 0.142 |  | 1.18(0.97-1.45) | 0.106 |
| Smoking history: | |  |  |  |  |  |
| Ever Smoker | 6.9(6.0-7.8) | 0.88(0.73-1.06) | 0.164 |  | 0.94(0.71-1.25) | 0.671 |
| Non-smoker | 5.7(4.7-6.8) |  |  |  |  |  |
| ECOG PS: |  |  |  |  |  |  |
| 1 | 6.6(5.9-7.3) |  |  |  |  |  |
| 2 | 4.4(2.3-6.5) | 1.51(1.10-2.07) | 0.011 |  | 1.50(1.09-2.06) | 0.012* |
| TNM |  |  |  |  |  |  |
| IIIa | 9.0(7.4-10.7) |  |  |  |  |  |
| IIIb | 6.0(4.4-7.6) |  |  |  |  |  |
| IV | 6.3(5.5-7.3) | 1.10(0.95-1.26) | 0.213 |  | 1.09(0.94-1.26) | 0.268 |
| Histology |  |  | 0.714 |  |  | 0.796 |
| adenocarcinoma | 6.4(5.5-7.4) | 1 |  |  | 1 |  |
| squamous cell | 6.9(5.4-8.4) | 0.91(0.72-1.15) | 0.433 |  | 0.98(0.75-1.27) | 0.871 |
| adenosquamous | 6.6(2.2-11.1) | 1.11(0.55-2.23) | 0.780 |  | 1.26(0.56-2.87) | 0.580 |
| others | 6.2(4.6-7.8) | 0.87(0.65-1.17) | 0.361 |  | 0.88(0.66-1.19) | 0.421 |
| rs3829254 | recessive |  |  |  |  |  |
| A/A | 7.0(6.0-7.9) |  |  |  |  |  |
| A/G+G/G | 5.1(3.9-6.3) | 1.27(1.04-1.54) | 0.015* |  | 1.16(0.91-1.48) | 0.225 |
| rs3910384 | dominate |  |  |  |  |  |
| A/A+A/G | 5.3(4.3-6.2) |  |  |  |  |  |
| G/G | 8.6(7.0-10.2) | 0.74(0.61-0.90) | 0.002* |  | 0.62(0.48-0.80) | 3.2E-4* |
| rs4370932 | additive |  |  |  |  |  |
|  | NA | 1.03(0.91-1.17) | 0.636 |  | 0.83(0.70-1.00) | 0.052 |
| rs1049403 | recessive |  |  |  |  |  |
| A/A | 7.0(6.0-7.9) |  |  |  | 0.90(0.59-1.37) | 0.608 |
| G/G+A/G | 5.1(3.9-6.4) | 1.25(1.03-1.51) | 0.025* |  |  |  |

**p*<0.05. Variables used in the multivariate analysis: Age, Gender, smoking history, ECOG PS, TNM stage, histology, the rs3829254 recessive model, the rs3910384 dominant model, the rs4370932 additive model and the rs1049403 recessive model.

**Supplementary Table S5. Kaplan-Meier overall survival analysis of WEE1 rs3910384 genotype stratified by combination chemotherapy regimen.**

| rs3910384  dominate model |  | mOS (95% CI) | | X^2^ | *p* |
| --- | --- | --- | --- | --- | --- |
|  |  | G/G | A/A+A/G |  |  |
| DNA damaging agents |  | 28.2(20.5-35.9) | 14.7(12.8-16.6) | 16.53 | 4.8E-5* |
| Tubulin-targeting drugs |  | 19.1(16.6-21.6) | 17.5(15.3-19.8) | 1.48 | 0.223 |
| other combination |  | 17.4(0-35.3) | 18.4(10.0-26.8) | 0.99 | 0.321 |

**p*<0.05.

**Supplementary Table S6. The 1, 2, 3-year survival rates of the 152 NSCLC patients treated with platinum-gemcitabine regimen.**

| rs3910384 A/A+G/G | |  | rs3910384 G/G | | *p* |
| --- | --- | --- | --- | --- | --- |
| life point | survival rate |  | life point | survival rate |
| 1-year | 0.221 |  | 1-year | 0.543 | 3.5E-04* |
| 2-year | 0.147 |  | 2-year | 0.388 |
| 3-year | 0.074 |  | 3-year | 0.277 |

**p*<0.05.

**Supplementary Table S7. Cox's regression analysis of prognostic factors for PFS in the 152 NSCLC patients treated with platinum-gemcitabine regimen.**

|  | mPFS(95%CI) | Univariate | | |  | | Multivariate | | | |
| --- | --- | --- | --- | --- | --- | --- | --- | --- | --- | --- |
|  |  | Hazard ratio (95% CI) | *p* | |  | | Hazard ratio (95% CI) | | *p* | |
| Age |  |  |  | |  | |  | |  | |
| ≥60 | 7.0(3.3-10.7) |  |  | |  | |  | |  | |
| ＜60 | 6.0(4.1-7.8) | 1.05(0.72-1.54) | 0.797 | |  | | 1.04(0.69-1.57) | | 0.858 | |
| Gender: |  |  |  | |  | |  | |  | |
| Male | 5.3(3.3-7.3) |  |  | |  | | 1.05(0.55-2.02) | | 0.889 | |
| Female | 7.2(3.5-11.0) | 0.86(0.59-1.27) | 0.457 | |  | |  | |  | |
| Smoking history: | |  |  | |  | |  | |  | |
| Never smoker | 6.7(4.9-8.5) |  |  | |  | |  | |  | |
| Ever Smoker | 4.8(1.3-8.3) | 1.09(0.74-1.59) | | 0.676 | |  | | 1.17(0.76-1.82) | | 0.475 |
| ECOG PS: |  |  |  | |  | |  | |  | |
| 1 | 6.6(4.8-8.5) |  |  | |  | |  | |  | |
| 2 | 3.7(0.8-6.5) | 2.37(1.13-4.95) | 0.022* | |  | | 2.05(0.97-4.32) | | 0.059 | |
| TNM |  |  |  | |  | |  | |  | |
| IIIa | 9.2(4.5-13.8) |  |  | |  | |  | |  | |
| IIIb | 5.4(1.7-9.2) |  |  | |  | |  | |  | |
| IV | 6.0(4.3-7.7) | 1.16(0.87-1.56) | 0.316 | |  | | 1.23(0.88-1.70) | | 0.223 | |
| Histology |  |  | 0.205 | |  | |  | | 0.295 | |
| adenocarcinoma | 7.2(4.6-9.8) | 1 |  | |  | | 1 | |  | |
| squamous | 4.4(3.6-5.3) | 1.64(0.97-2.75) | 0.063 | |  | | 1.60(0.94-2.73) | | 0.083 | |
| adenosquamous | 1.1(NA) | 2.4(0.59-9.86) | 0.224 | |  | | 2.07(0.50-8.61) | | 0.316 | |
| others | 4.0(0-8.7) | 1.12(0.56-2.24) | 0.750 | |  | | 1.10(0.55-2.20) | | 0.796 | |
| rs3829254 | recessive |  |  | |  | |  | |  | |
| A/A | 7.6(5.0-10.1) |  |  | |  | |  | |  | |
| A/G+G/G | 4.4(2.9-5.9) | 1.39(0.94-2.05) | 0.097 | |  | | 1.69(0.59-4.79) | | 0.328 | |
| rs3910384 | dominate |  |  | |  | |  | |  | |
| G/G | 9.2(5.8-12.6) | 0.60(0.40-0.89) | 0.012* | |  | | 0.63(0.42-0.94) | | 0.023* | |
| A/A+A/G | 4.9(2.9-6.9) |  |  | |  | |  | |  | |
| rs4370932 | additive |  |  | |  | |  | |  | |
|  | NA | 1.22(0.92-1.61) | 0.171 | |  | | 0.86(0.58-1.29) | | 0.464 | |
| rs1049403 | recessive |  |  | |  | |  | |  | |
| A/A | 7.6(4.2-10.9) |  |  | |  | | 0.82(0.48-1.39) | | 0.454 | |
| G/G+A/G | 4.6(3.0-6.1) | 1.30(0.88-1.93) | 0.183 | |  | |  | |  | |

**p*<0.05. Variables used in the multivariate analysis: Age, Gender, smoking history, ECOG PS, TNM stage, histology, the rs3829254 recessive model, the rs3910384 dominant model, the rs4370932 additive model and the rs1049403 recessive model.

**Supplementary Table S8. Multivariant Cox regession analysis of prognostic factors of OS in validation cohort.**

| Variables | | | N = 264 | mOS (95%CI) | HR (95% CI) | *p* |
| --- | --- | --- | --- | --- | --- | --- |
| Median age, y (range) | | |  |  |  |  |
| <60 | | | 142(53.8%) | 22.3(18.8-25.8) | 1.32(0.92-1.90) | 0.136 |
| ≥60 | | | 122(46.2%) | 15.8(10.6-21.1) |  |  |
| Gender | | |  |  |  |  |
| Male | | | 194(73.5%) | 19.6(16.1-23.2) | 0.68(0.43-1.09) | 0.111 |
| Female | | | 70(26.5%) | 26.9(14.4-39.4) |  |  |
| Smoking history | | |  |  |  |  |
| Nonsmoker | | | 112(43.1%) | 23.9(16.8-30.9) | 0.87(0.51-1.48) | 0.610 |
| Ever Smoker | | | 148(56.9%) | 19.9(14.8-25.0) |  |  |
| ECOG PS | | |  |  |  |  |
| 0 | | | 8(3%) | 13.1(1.4-24.8) |  |  |
| 1 | | | 231(87.5%) | 20.9(17.9-23.9) | 1.24(0.73-2.12) | 0.430 |
| 2 | | | 25(9.5%) | 11.8(0-23.2) |  |  |
| Tumor histology | | |  |  |  |  |
| Adenocarcinoma | | | 154(59.0%) | 19.9(16.3-23.5) | 1 | 0.275 |
| Squamous cell | | | 62(23.8%) | 23.3(18.7-28.0) | 0.65(0.41-1.03) | 0.067 |
| Adenosquamous cell | | | 4(1.5%) | 22.0(ND) | 0.45(0.06-3.30) | 0.429 |
| Other NSCLC | | | 41(15.7%) | 20.4(11.8-29.0) | 0.80(0.46-1.38) | 0.419 |
| TNM | | |  |  |  |  |
| IIIa | | | 27(10.3%) | 23.6(9.6-37.5) | 1.09(0.82-1.44) | 0.557 |
| IIIb | | | 83(31.8%) | 21.7(20.0-23-4) |  |  |
| IV | | | 151(57.9%) | 19.4(16.8-22.1) |  |  |
| rs3829254 | | |  |  |  |  |
| recessive | AA | | 186(71.0%) | 21.5(19.0-24.0) | 0.84(0.50-1.40) | 0.507 |
|  | GG and AG | | 76(29.0%) | 17.2(9.3-25.0) |  |  |
| rs3910384 | | |  |  |  |  |
| dominant | AA and AG | | 137(51.9%) | 15.9(11.6-20.2) | 0.41(0.25-0.67) | 3.9E-4* |
|  | GG | | 127(48.1%) | 23.9(18.8-28.9) |  |  |
| rs4370932 | | |  |  |  |  |
| additive | | | NA | NA | 0.68(0.49-0.94) | 0.019* |
| rs1049403 | | |  |  |  |  |
| recessive | | AA | 186(70.7%) | 21.8(18.6-25.0) | 2.49(0.78-7.94) | 0.123 |
|  |  | GG and AG | 77(29.3%) | 17.2(11.7-22.6) |  |  |

**p*<0.05. Variables used in the multivariate analysis: Age, Gender, smoking history, ECOG PS, TNM stage, histology, the rs3829254 recessive model, the rs3910384 dominant model, the rs4370932 additive model and the rs1049403 recessive model.

**Supplementary Table S9. Rs3910384 allele-based kaplan-meier overall survival analysis for overall survival in the 152 NSCLC patients treated with platinum-gemcitabine regimen.**

| rs3910384 | N=152 | mOS(95%CI) | HR (95% CI) | *p* |
| --- | --- | --- | --- | --- |
| G/G | 63 | 28.2(20.5-35.9) | 1 | 1.79E-4 (G/G vs. A/G) |
| A/A | 17 | 14.4(5.7-23.2) | 2.86(1.48-5.52) | 1.57E-3 (A/A vs. G/G) |
| A/G | 71 | 15.0(12.8-17.1) | 2.27(1.46-3.51) | 4.81E-1 (A/G vs. A/A) |

**Supplementary Figure S1.** **Kaplan-Meier curve of overall survival (OS) according to polymorphisms of *WEE1* tag SNPs.**


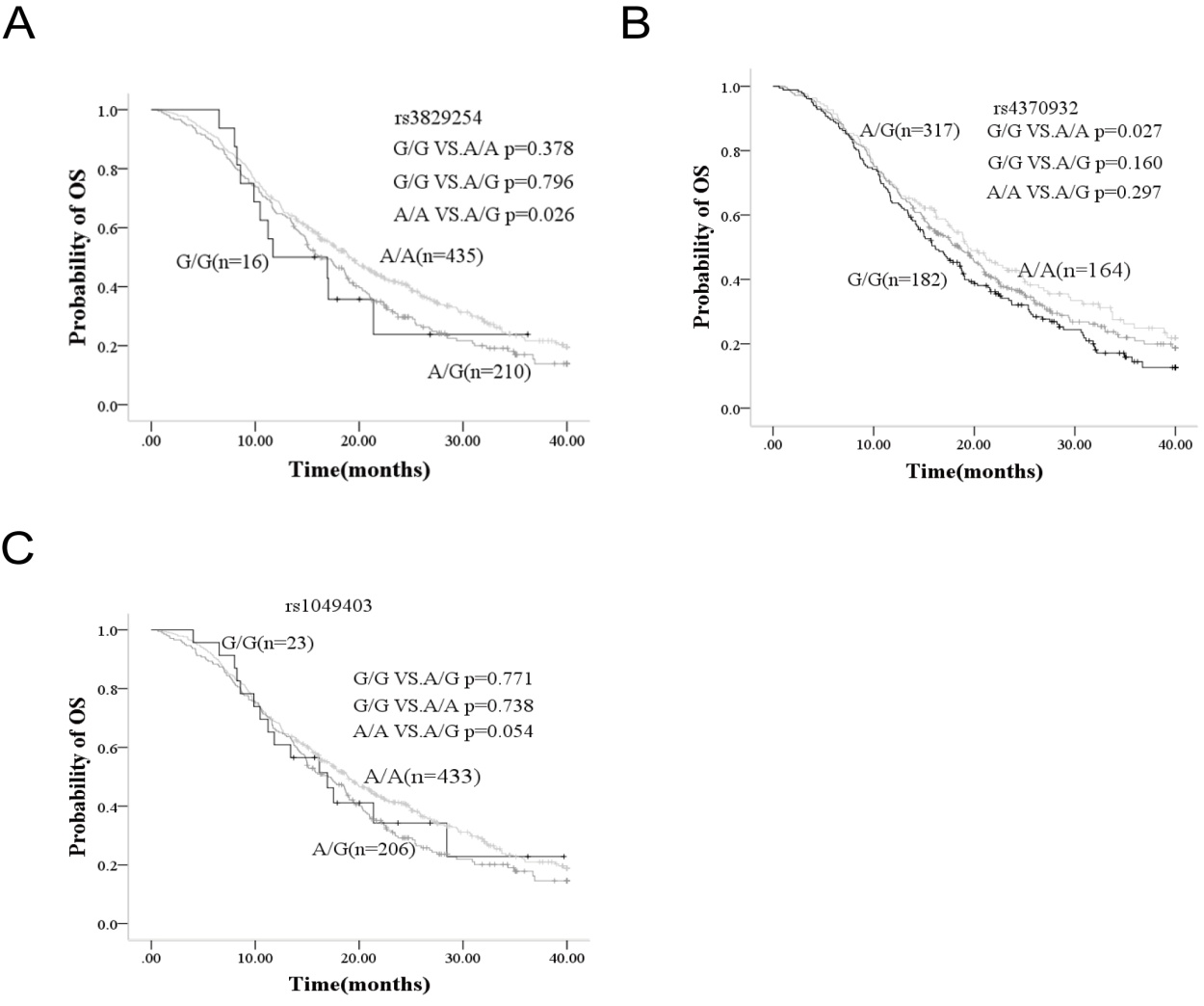


**A**: Kaplan-Meier curve of OS for rs3829254 in patients with NSCLC.

**B**: Kaplan-Meier curve of OS for rs4370932 in patients with NSCLC.

**C**: Kaplan-Meier curve of OS for r1049403 in patients with NSCLC.

**Supplementary Figure S2. Kaplan-Meier curve of progression free survival (PFS) according to polymorphisms of *WEE1* tag SNPs.**


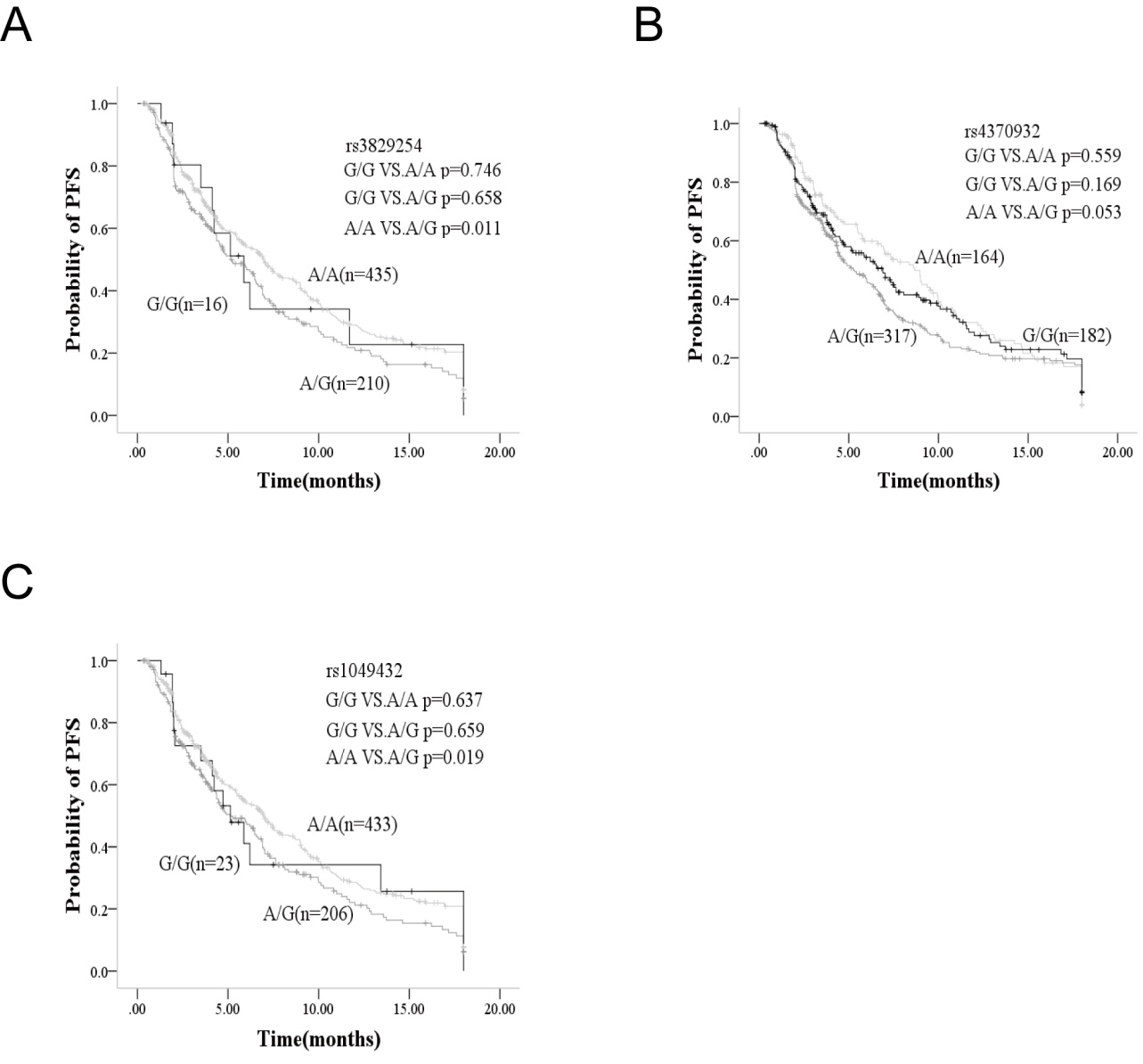


**A**: Kaplan-Meier curve of PFS for rs3829254 in patients with NSCLC.

**B**: Kaplan-Meier curve of PFS for rs4370932 in patients with NSCLC.

**C**: Kaplan-Meier curve of PFS for r1049403 in patients with NSCLC.
